# The impact of the COVID-19 pandemic and stringent social distancing measures on health-related quality of life and COVID-19 infection rates in patients with rheumatic disease: a longitudinal analysis through the pandemic

**DOI:** 10.1093/rap/rkad009

**Published:** 2023-01-16

**Authors:** Natasha Cox, Sabrina R Raizada, Nick Barkham, Srinivasan Venkatachalam, Tom P Sheeran, Tochukwu Adizie, Hem Sapkota, Ian C Scott, Sara Muller, James Bateman

**Affiliations:** Primary Care Centre Versus Arthritis, School of Medicine, Keele University, Keele, Newcastle-under-Lyme, UK; The Royal Wolverhampton NHS Trust, Wolverhampton, UK; The Royal Wolverhampton NHS Trust, Wolverhampton, UK; The Royal Wolverhampton NHS Trust, Wolverhampton, UK; The Royal Wolverhampton NHS Trust, Wolverhampton, UK; The Royal Wolverhampton NHS Trust, Wolverhampton, UK; The Royal Wolverhampton NHS Trust, Wolverhampton, UK; Primary Care Centre Versus Arthritis, School of Medicine, Keele University, Keele, Newcastle-under-Lyme, UK; Primary Care Centre Versus Arthritis, School of Medicine, Keele University, Keele, Newcastle-under-Lyme, UK; The Royal Wolverhampton NHS Trust, Wolverhampton, UK; Institute of Clinical Sciences, College of Medical and Dental Sciences, University of Birmingham, Birmingham, UK

**Keywords:** health-related quality of life, patient outcomes, rheumatic disease, stringent social isolation, SMS communications, health policy

## Abstract

**Objective:**

The aim was to evaluate the impact of the coronavirus disease 2019 (COVID-19) pandemic and stringent social isolation measures on patients with rheumatic disease (RD) from the beginning of the pandemic (April 2020).

**Methods:**

In this UK-based single-centre, prospective, observational cohort study, all RD follow-up patients at our centre were invited by SMS text message in April 2020 to participate in the study. Participants completed questionnaires at four time points between April 2020 and December 2021. We collected demographics, clinically extremely vulnerable (CEV) status, short form 12 mental (MCS) and physical health component scores (PCS) for health-related quality of life, vaccination status, COVID-19 infection rates and incidence of long COVID.

**Results:**

We enrolled 1605 patients (female, 69.0%; CEV, 46.5%); 906 of 1605 (56.4%) completed linked responses to our final questionnaire. MCS improved (+0.6, *P* < 0.05), whereas PCS scores deteriorated (−1.4, *P* < 0.001) between April 2020 and December 2021. CEV patients had worse mental and physical health scores than non-CEV patients at entry (PCS, 36.7 and 39.3, respectively, *P* < 0.001; MCS, 40.9 and 43.0, respectively, *P* < 0.001) and at each time point throughout the study; both mental and physical health outcomes were worse in CEV compared with non-CEV patients (*P* < 0.001 and *P* = 0.004, respectively). At study close, 148 of 906 (16.3%) reported COVID infection, with no difference in infection, vaccination or long COVID rates between CEV and non-CEV patients.

**Conclusions:**

Mental and physical health in RD patients has changed throughout the pandemic; outcomes for both metrics of health were worse in CEV patients, although there were no differences in infection rates between the groups. These data might assist the understanding and planning of future health-care policy and social restrictions in RD patients.

**Trial registration:**

ClinicalTrials.gov, www.clinicaltrials.gov, NCT04542031.

Key messages

- Clinically Extremely Vulnerable (CEV) patients had worse mental and physical health outcomes over the course of the pandemic.
- There is no difference in COVID infection or long COVID rates between our CEV and non-CEV groups despite extra precautionary measures in our CEV group.
- Novel SMS-based messaging can invite large cohorts of patients to research studies remotely, at low cost.

## Introduction

The coronavirus disease 2019 (COVID-19) pandemic has had unprecedented effects worldwide, with countries adopting differing approaches to social lockdown to mitigate the spread of the virus. In the UK, national social distancing measures were implemented to varying degrees between March 2020 and December 2021 encompassing periods of complete national lockdown that required people to remain in their homes unless it was absolutely necessary to leave [1]. During the early stages of the pandemic, national policy deemed many patients with rheumatic disease (RD) to be at higher risk from COVID-19, and those RD patients with strong immunosuppressive therapies and co-morbidity were categorized as clinically extremely vulnerable (CEV) [2]. These patients were required to follow stringent social distancing measures as part of a national ‘shielding’ programme aimed at protecting those most vulnerable in society [3]. The shielding programme started in mid-March 2020 and involved additional precautionary measures in CEV individuals during periods of high infection rates in the general population, including a period of extreme social isolation between 23 March 2020 and 31 July 2020. As vaccines for SARS-CoV-2 were developed and increasing numbers of the general and CEV population were protected by immunization, distancing restrictions lessened, and the shielding programme was paused in April 2021 and did not restart before being ended formally in September 2021 [4].

It is recognized that patients with RD have poorer baseline physical and mental health than the general population, and studies during the first wave of the pandemic highlighted the negative impacts this stringent social isolation guidance had on both physical and psychological wellbeing in RD patients during these initial stages [5–11]. Studies assessing the medium- and longer-term impacts of stringent social isolation guidance in RD patients are lacking. One study recruiting via social media has explored the impacts of social isolation measures on mental and physical health in patients with RD past the initial stages of the pandemic to November 2020 and suggests that although social isolation measures in RD patients were initially detrimental, the negative effects tapered as the pandemic progressed, and both mental and physical health started to recover by November 2020. However, those authors acknowledge the selection bias related to social media recruitment and the challenges of following patients over time [10]. Further investigation exploring the impact of stringent distancing measures in RD patients on mental and physical wellbeing spanning the duration of lockdown measures using robust recruitment strategies are required.

Mobile communication and tele-rheumatology advances during the pandemic have transformed approaches to RD care, including the rapid distribution of short messaging service (SMS) and smartphone video advice and national capturing of electronic patient-reported outcome measures (ePROMs) using the British Society for Rheumatology’s ePROMs platform [12–14]. The primary aim of the COVID-19 Rheumatology Impact and Surveillance Project (CRISP) study was to build on these tele-rheumatology advances by applying an innovative SMS-based recruitment strategy and data collection to determine the impact of COVID-19 pandemic social distancing (lockdown) requirements on physical and mental health-related quality of life (HRQoL) in patients with RD between April 2020 and December 2021. Its secondary aim was to evaluate these social distancing requirements on self-reported COVID-19 infection rates.

## Methods

### Study design

In this prospective longitudinal observational study, all rheumatology follow-up patients at the Royal Wolverhampton Trust were sent an SMS invitation to participate in the study (clinical trial number: NCT04542031). The SMS message included links to the participant information sheet and a consent form that was completed by all study participants. Study inclusion criteria comprised patients with RDs, aged ≥18 years, under follow-up at the Royal Wolverhampton Trust, with a verified mobile telephone number linked to their electronic health care record on 24 April 2020, responding within 7 days of the invitation, who had not opted out of SMS-based messaging. We have previously reported the methodology [9].

**Figure 1.**
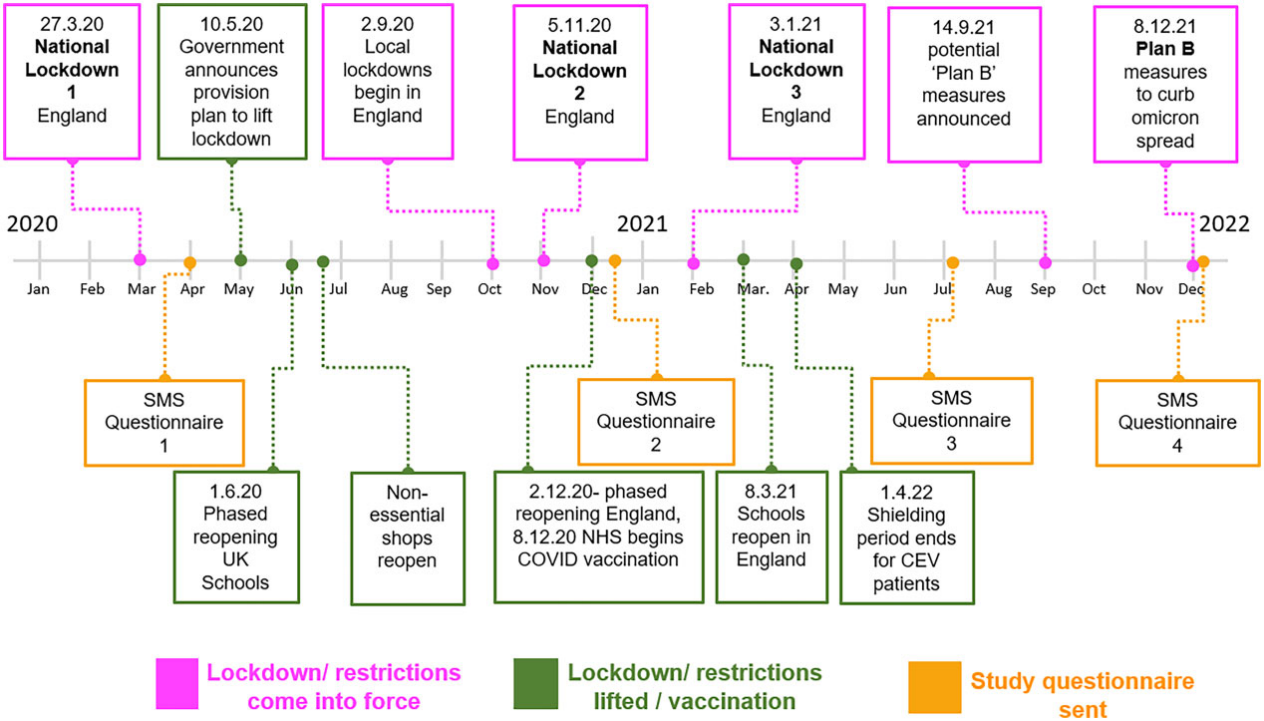
Schematic diagram of the coronavirus disease 2019 (COVID-19) lockdown in England, highlighting the timing of the study questionnaires

Data were collected using web-based questionnaires (Momentive, formerly SVMK, 2020). Questionnaires were distributed via our existing SMS provider (Healthcare Communications UK) at two time points (April 2020 and December 2021), with interim monitoring questionnaires distributed in December 2020 and June 2021. A time line showing the timing of the questionnaires and the COVID-19 social distancing measures is shown in Fig. 1. After distribution of each questionnaire, participants were sent an SMS reminder. For each questionnaire, data collection closed at the 4-week point after distribution. Participants could only submit completed questionnaires, with prompts for missing answers.

During the study, all patients in our cohort, irrespective of participation, received SMS-based video advice on shielding stratification (March 2020), making them aware of their CEV status and whether they needed to shield, and a video promoting safety of the COVID-19 vaccination (December 2020) as part of their usual care.

### Patient and public involvement

Two patient participation groups and a national charity (Hibbs Lupus Trust) were involved in the design and piloting of the Web-based data collection tool.

### Outcome measures

We designed and piloted a 70-item questionnaire including: self-reported demographics; CEV group status requiring ‘shielding’, adherence to ‘shielding’, vaccination status, and physical and mental health assessed by the short form 12 (SF-12) questionnaire version 1, self-reported COVID-19 infection and symptoms if infected, and the impact of video-based health advice sent to participants (the final questionnaire released in December 2021 is provided in Supplementary Data S1, available at *Rheumatology Advances in Practice* online). SF-12 scores were summarized as physical (PCS) and mental component scores (MCS) on a 0–100 scale (0 = lowest quality of health) [15, 16]. The Web-based questionnaire was piloted and reviewed by clinicians and our patient participation group. Ethnicity was self-reported from a predetermined list as White or non-White groups. The presence of long COVID was defined as self-reported symptoms persisting for >4 weeks, encompassing ‘ongoing symptoms’ (symptoms >4 weeks, <12 weeks) and ‘post-COVID syndrome’ (12 weeks or more) [17].

### Statistical analysis

Study variables were analysed descriptively in each questionnaire (frequency and percentage for count data; mean and s.d. for continuous data). Age, gender and ethnicity were also reported, stratified by CEV status.

Student’s unpaired *t*-tests were used to assess for differences in mean MCS and PCS scores between CEV and non-CEV groups in each questionnaire. The relationship between CEV status and change in PCS and MCS scores between questionnaires 1 and 4 were evaluated using regression models. These included questionnaire 4 PCS or MCS scores as the response variable and CEV status and questionnaire 1 PCS or MCS scores as the explanatory variables, adjusted for the confounding variables (age, ethnicity, gender and COVID-19 infection during the study period).

COVID-19 infection rates and shielding behaviours between CEV and non-CEV groups were compared using chi-squared tests. In addition, a logistic regression model further examined the relationship between infection rates and CEV status. This included previous self-reported COVID-19 infection at questionnaire 4 as the response variable and CEV status as the explanatory variable.

The significance level for all analysis was a *P*-value < 0.05, and data were analysed using the IBM SPSS v.27.0 (IBM Corp., Armonk, NY, USA).

### Ethical approval

This study involves human participants. The initial questionnaire in April 2020 was approved by the Royal Wolverhampton Trust senior management group as part of a service evaluation. The trial was granted institutional review board ethics approval by London—Brent Research Ethics Committee [HRA number: 20/HRA/4882]. Participants gave informed consent to participate in the study before taking part.

## Results

**Figure 2.**
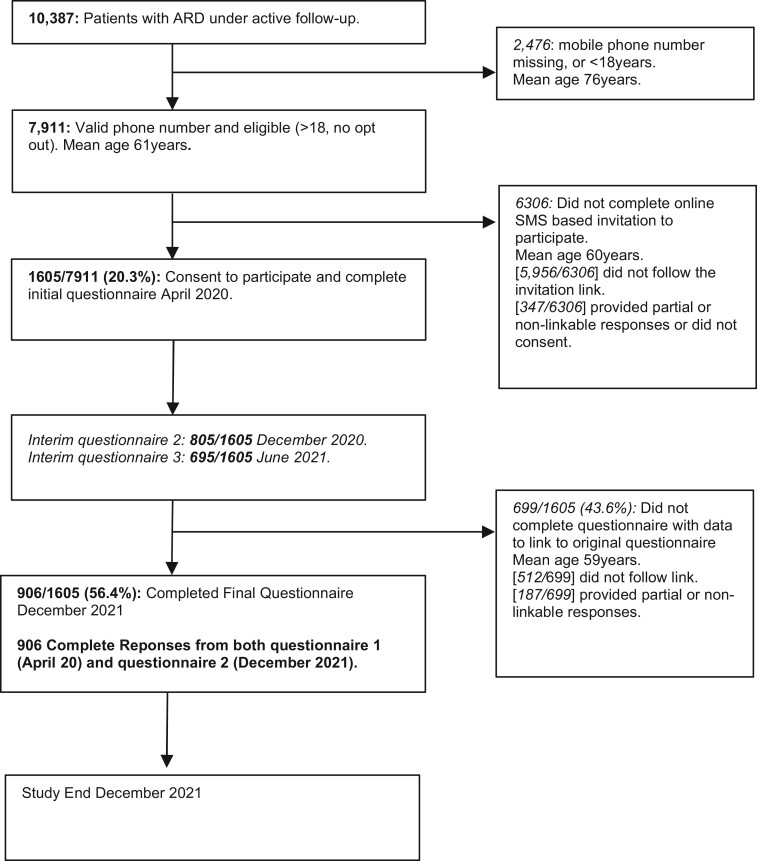
Enrolment and follow-up to our study

By 24 April 2020, there were 10 387 rheumatology patients under active rheumatology follow-up; 7911 (76.2%) had validated mobile telephone numbers on record. At data collection, 1694 (21.4%) patients had responded to questionnaire one; 1636 of 1694 (96.6%) of these participants agreed to participate in the longitudinal study, and 1605 of 1636 (98.1%) provided complete responses to all aspects of the questionnaire. Of these 1605 individuals, complete responses that were able to be linked to questionnaire 1 were received from 805 (50.2%) for questionnaire 2, 695 (43.3%) for questionnaire 3 and 906 (56.4%) for questionnaire 4. The study participant flow, with mean ages, is presented in Fig. 2.

### Demographics

Demographics were similar across the four questionnaires. Responders were primarily female (ranging from 68.7 to 69.1% across the questionnaires), White (93.8–96.4%) and with mean ages between 61 and 63 years. The majority had inflammatory arthritis (RA, 49.5–52.1%; PsA, 14.4–16.0%; and AS, 3.7–5.1%); between 33.3 and 38.4% were on conventional DMARDs, 32.1–34.4% were on biologic DMARDs and 11.7–12.3% were on CSs across the questionnaires (Table 1). Of the responders, 746 (46.5%), 379 (47.1%), 319 (45.9%) and 428 (47.2%) were classed as CEV in questionnaires 1, 2, 3 and 4, respectively.

**Table 1.** Demographics, coronavirus disease 2019 infection, clinically extremely vulnerable status and health-related quality of life data

Parameter	April 2020 (questionnaire 1), *n* (%)	December 2020 (questionnaire 2), *n* (%)	June 2021 (questionnaire 3), *n* (%)	December 2021 (questionnaire 4), *n* (%)
*n*	1605	805	695	906
Gender				
Male	498 (31.0)	249 (30.9)	215 (30.9)	284 (31.3)
Female	1107 (69.0)	556 (69.1)	480 (69.1)	622 (68.7)
Ethnicity				
White	1506 (93.8)	769 (95.5)	670 (96.4)	865 (95.5)
Non-White	99 (6.2)	36 (4.5)	25 (3.6)	41 (4.5)
Diagnosis				
RA	794 (49.5)	420 (52.2)	357 (51.4)	469 (51.8)
PsA	257 (16.0)	116 (14.4)	109 (15.7)	141 (15.6)
AS	81 (5.0)	37 (4.6)	26 (3.7)	35 (3.9)
SLE	40 (2.5)	16 (2.0)	12 (1.7)	24 (2.6)
OA	95 (5.9)	46 (5.7)	36 (5.2)	51 (5.6)
Vasculitis	14 (0.9)	10 (1.2)	8 (1.2)	9 (1.0)
Myositis/DM	11 (0.7)	5 (0.6)	2 (0.3)	4 (0.4)
Scleroderma/SSc	21 (1.3)	7 (0.9)	7 (1.0)	11 (1.2)
Osteoporosis	77 (4.8)	40 (5.0)	42 (6.0)	48 (5.3)
Other	215 (13.4)	108 (13.4)	96 (13.8)	114 (12.6)
Age				
<60 years	792 (49.3)	335 (41.6)	290 (41.7)	387 (42.7)
≥60 years	813 (50.7)	470 (58.4)	405 (58.3)	519 (57.3)
Years, mean (SD)	61 (12.3)	63 (11.3)	63 (10.8)	63 (11.0)
Medication				
cDMARD	535 (33.3)	308 (38.3)	267 (38.4)	334 (36.9)
bDMARD ± cDMARD	516 (32.1)	272 (33.8)	234 (33.7)	312 (34.4)
Prednisolone				
<10 mg/day	147 (9.2)	78 (9.7)	62 (8.9)	90 (9.9)
10–19 mg/day	39 (2.4)	17 (2.1)	15 (2.2)	15 (1.7)
>20 mg/day	8 (0.5)	4 (0.5)	4 (0.6)	3 (0.3)
Self-reported COVID infection (since the last questionnaire)	60 (3.7)	33 (4.1)	15 (2.2)	101 (11.1)
CEV group				
*n*	746 (46.5)	379 (47.1)	319 (45.9)	428 (47.2)
Age ≥60 years	435 (58.3)	243 (64.1)	199 (62.4)	266 (62.1)
Female	519 (69.6)	272 (71.8)	222 (69.6)	300 (70.1)
Non-White	55 (7.4)	19 (5.0)	16 (5.0)	28 (6.5)
Non-CEV group				
*n*	859 (53.5)	426 (52.9)	376 (54.1)	478 (52.8)
Age ≥60 years	378 (44.0)	227 (53.3)	206 (54.8)	253 (52.9)
Female	588 (68.5)	284 (66.7)	258 (68.6)	322 (67.4)
Non-White	44 (5.1)	16 (3.8)	9 (2.4)	13 (2.7)

bDMARD: biologic DMARD; cDMARD: conventional DMARD.

### Health-related quality of life

In all questionnaires, MCS scores were significantly lower in CEV than non-CEV participants: April 2020, mean MCS 40.9 (s.d. 7.4) and 43.0 (s.d. 6.9), *P* <* *0.001, CEV and non-CEV, respectively; December 2020, 41.3 (s.d. 7.6) and 43.7 (s.d. 6.7), *P* <* *0.001; June 2021, 40.8 (s.d. 7.0) and 43.1 (s.d. 6.8), *P* <* *0.001; and December 2021, 41.5 (s.d. 7.2) and 43.6 (s.d. 6.4), *P* <* *0.001.

Between questionnaires 1 and 4, MCS scores improved significantly by a mean of +0.6 units (95% CI 0.02, 1.18), *P* =* *0.042, in all individuals. In a linear regression model controlling for MCS at baseline (April 2020), age, ethnicity, gender and COVID-19 status, the found outcome MCS was significantly worse in the CEV group compared with non-CEV group [β = −1.34 (95% CI −2.16, −0.51); *P* <* *0.001; Table 2].

**Table 2.** Health-related quality of life scores in the entire cohort and comparing CEV and non-CEV groups

	Health-related quality of life scores, mean (s.d.)			
Short form 12 score, mean (SD)	Whole group	CEV	Non-CEV	*P*-value
MCS scores				
April 2020	42.0 (7.2)	**40.9 (7.4)**	**43.0 (6.9)**	**<0.001**
December 2020	42.5 (7.3)	**41.3 (7.6)**	**43.7 (6.7)**	**<0.001**
June 2021	42.0 (7.0)	**40.8 (7.0)**	**43.1 (6.8)**	**<0.001**
December 2021	42.6 (6.9)	**41.5 (7.2)**	**43.6 (6.4)**	**<0.001**
Change over time* (95% CI)	**+0.6 (0.02, 1.18)**	**+0.6 (0.03, 1.20)**	**+0.6 (0.01, 1.18)**	**<0.001**
β (95% CI)		**−1.34 (−2.16, −0.51)**		
PCS scores				
April 2020	38.4 (6.6)	**36.7 (6.7)**	**39.8 (6.3)**	**<0.001**
December 2020	37.4 (6.3)	**36.1 (6.2)**	**38.5 (6.1)**	**<0.001**
June 2021	36.7 (6.2)	**35.9 (6.0)**	**37.4 (6.4)**	**0.002**
December 2021	37.0 (6.1)	**35.7 (6.0)**	**38.1 (6.0)**	**<0.001**
Change over time* (95% CI)	**−1.4 (0.88, 1.92)**	**−1.0 (−0.37, −1.43)**	**−1.7 (−1.20, −2.20)**	**0.004**
β (95% CI)		**−1.08 (−1.81, −0.35)**		

Health-related quality of life scores are compared at each time point with linear regression models assessing the impact of CEV status on the outcome health-related quality of life by December 2021, while controlling for baseline and confounding factors. Significant results are in bold. Significance was measured to *P* < 0.05.

β: unstandardized beta; CEV: clinically extremely vulnerable; MCS: mental component score; PCS: physical component score.

*Change between April 2020 and December 2021.

Likewise, in all questionnaires the PCS scores were significantly lower in the CEV group than in non-CEV participants at all time points: April 2020, mean 36.7 (s.d. 6.7) *vs* 39.8 (s.d. 6.3), *P* <* *0.001; December 2020, 36.1 (s.d. 6.2) *vs* 38.5 (s.d. 6.1), *P* <* *0.001; June 2021, 35.9 (s.d. 6.0) *vs* 37.4 (s.d. 6.4), *P* =* *0.002; and December 2021, 35.7 (s.d. 6.0) *vs* 38.1 (s.d. 6.0), *P* <* *0.001.

Between questionnaires 1 and 4, PCS scores declined significantly by a mean of −1.4 units (95% CI 0.88, 1.92), *P* <* *0.001, in all individuals. In a linear regression model controlling for confounding factors, the found outcome PCS was significantly worse in CEV compared with non-CEV participants [β −1.08 (95% CI −1.81, −0.35), *P* =* *0.004] compared with baseline (Table 2).

### COVID-19 infection and vaccination rates

Self-reported new COVID-19 infection rates at each time point (since the previous questionnaire) are reported in Table 1. As would be expected, these increased across questionnaires from 3.7% in April 2020 to 11.1% December 2021. Overall COVID-19 infection rates over the 20-month period, comparing the CEV and non-CEV groups that completed questionnaire 4, are shown in Table 3.

**Table 3.** Coronavirus disease 2019 infection data

	Total, *n* (%)	CEV, *n* (%)	Non-CEV, *n* (%)
Self-reported COVID-19 infection	148/906 (16.3)	73/428 (17.1)	75/478 (15.7)
Acute COVID			
<24 h	3 (2.0)	2 (2.7)	1 (1.3)
1–2 days	7 (4.7)	5 (6.8)	2 (2.7)
3–7 days	38 (25.7)	9 (12.3)	29 (38.7)
1–2 weeks	29 (19.6)	21 (28.8)	8 (10.7)
2–3 weeks	21 (14.2)	6 (8.2)	15 (20.0)
3–4 weeks	14 (9.5)	8 (11.0)	6 (8.0)
Long COVID			
4–12 weeks	18 (12.2)	13 (17.8)	5 (6.7)
>12 weeks	18 (12.2)	9 (12.3)	9 (12.0)
Fever	59 (39.9)	34 (46.6)	25 (33.3)
Cough	49 (33.1)	35 (47.9)	24 (32.0)
Fatigue	128 (86.5)	65 (89.0)	63 (84.0)
Headache	116 (78.4)	55 (75.3)	61 (81.3)
Shortness of breath			
Mild	50 (33.8)	20 (27.4)	30 (40.0)
Significant	35 (23.6)	21 (28.8)	14 (18.7)
Severe	15 (10.1)	12 (16.4)	3 (4.0)
Sore throat	65 (43.9)	36 (49.3)	29 (38.7)
Loss of smell/taste	97 (65.5)	47 (64.4)	50 (66.7)
Abdominal pain	46 (31.1)	24 (32.9)	22 (29.3)
Hospital admission	18 (12.2)	10 (13.7)	8 (10.7)
Continued cDMARDs while infected			
Yes	27 (18.2)	13 (17.8)	19 (25.3)
No	46 (31.1)	27 (37.0)	11 (14.7)
Not taking cDMARDs	75 (50.7)	33 (45.2)	45 (60.0)
Continued bDMARDs while infected			
Yes	38 (25.7)	19 (26.0)	19 (25.3)
No	40 (27.0)	29 (39.7)	11 (14.7)
Not taking bDMARDs	70 (47.3)	25 (34.2)	45 (60.0)
Continued CSs while infected			
Yes	24 (16.2)	17 (23.3)	7 (9.3)
No	10 (6.8)	5 (6.8)	5 (6.7)
Not taking CSs	114 (77.0)	51 (69.9)	63 (84.0)
Received one or more COVID-19 vaccination by December 2021	903 (99.1)	423 (98.8)	475 (99.4)

Cumulative self-reported COVID-19 infection rates, infection characteristics, use of immunosuppressives during infection and vaccination status in those completing the final questionnaire, stratified by clinically extremely vulnerable status.

bDMARD: biologic DMARD; cDMARD: conventional DMARD; CEV: clinically extremely vulnerable; COVID-19: coronavirus disease 2019.

No statistically significant differences in COVID-19 infection rates (17.1 *vs* 15.7%; *P* =* *0.58) were observed between CEV and non-CEV groups. The duration of symptoms of infection [long COVID *vs* acute COVID, 22 (30.1%) *vs* 14 (18.7%)] and symptom types were similar between the groups, with the most common symptoms being fatigue (89.0 *vs* 84.0%) and headache (75.3 *vs* 81.3%) in CEV and non-CEV groups, respectively.

Vaccination rates were high in both cohorts at 99.1% overall. In December 2021, 423 (98.8%) in the CEV group and 475 (99.4%) in the non-CEV group self-reported having received one or more vaccine doses.

A logistic regression model assessing differences in COVID-19 infection rates between the CEV and non-CEV cohorts confirmed that there was no difference in infection rates between CEV and non-CEV groups [odds ratio 0.84 (0.57, 1.24), *P* =* *0.377].

### Self-isolation behaviours

Overall, a greater proportion of responders reported stricter self-isolation, having ‘not left the house’, in the week preceding questionnaire distribution in questionnaire 1 than in the subsequent questionnaires (31.8 *vs* 5.3, 3.0 and 4.4% for questionnaires 1, 2, 3 and 4, respectively; Table 4). By questionnaire 4, more were reporting ‘leaving the house often’ in the week preceding questionnaire release (42.5 *vs* 41.6, 25.0 and 8.8% for questionnaires 4, 3, 2 and 1, respectively).

**Table 4.** Comparison of isolation behaviours between clinically extremely vulnerable and non-clinically extremely vulnerable patients throughout the 20-month period

How much have you been self-isolating over the past week?	Total, *n* (%)	CEV, *n* (%)	Non-CEV, *n* (%)	Difference between groups, d.f. = 2 χ^2^, *P*-value
Questionnaire 1, *n* = 1605				
‘I have not left the house’	511 (31.8)	387 (51.9)	124 (14.4)	
‘I rarely leave the house’	953 (59.4)	347 (46.5)	606 (70.6)	
‘I leave the house often’	141 (8.8)	12 (1.6)	129 (15.0)	**298.35, *P* <* *0.001**
Questionnaire 2, *n* = 805				
‘I have not left the house’	43 (5.3)	32 (8.4)	11 (2.6)	
‘I rarely leave the house’	561 (69.7)	286 (75.5)	275 (64.6)	
‘I leave the house often’	201 (25.0)	61 (16.1)	140 (32.9)	**38.91, *P* <* *0.001**
Questionnaire 3, *n* = 695				
‘I have not left the house’	21 (3.0)	16 (5.0)	5 (1.3)	
‘I rarely leave the house’	385 (55.4)	201 (63.0)	184 (48.9)	
‘I leave the house often’	289 (41.6)	102 (32.0)	187 (49.7)	**27.02, *P* <* *0.001**
Questionnaire 4, *n* = 906				
‘I have not left the house’	40 (4.4)	22 (5.1)	18 (3.8)	
‘I rarely leave the house’	481 (53.1)	258 (60.3)	223 (46.7)	
‘I leave the house often’	385 (42.5)	148 (34.6)	237 (49.6)	**20.82, *P* <* *0.001**

CEV: clinically extremely vulnerable.

Self-isolation behaviours were significantly different between CEV and non-CEV participants at all time points. In all questionnaires, a greater proportion of the CEV group reported having ‘not left the house’ in the week before questionnaire distribution, whereas more in the non-CEV group reported having to ‘leave the house often’ in the week before questionnaire distribution (Table 4).

## Discussion

This single-centre longitudinal observational study is the first of its type to assess HRQoL scores systematically in rheumatology patients during the COVID-19 pandemic spanning April 2020 to December 2021. Although stringent social restrictions, social distancing and regional and national restrictions might impact the spread of the virus, we describe the impact of the measures on patients with rheumatic disease.

### Health-related quality of life

Patients in our CEV group, by definition, had a combination of risk factors for severe COVID-19 infection, including drug therapy (CSs, biologics, small molecules and traditional DMARDs), co-morbidity and age; and our data suggest that significantly more people in the CEV group exercised stringent social distancing throughout the pandemic compared with the non-CEV group [18]. Although numerous studies conducted during the early stages of the pandemic have suggested that, owing to a combination of stringent social isolation and the significant psychological burden that increased susceptibility to COVID-19 infection carries, those who are deemed CEV are at greater risk of worse outcomes during the COVID-19 pandemic, data suggest that the detrimental consequences of the pandemic in CEV patients with RD tapered as the pandemic progressed [10, 19, 20]. However, the mental health of CEV patients in our study remained significantly worse than that of the non-CEV group throughout the 20-month study. Although the mental health in the entire cohort improved between April 2020 and December 2021, which might reflect reassurance in the cohort, attributable, in part, to the remarkably high vaccination rates in all responders, shielding being paused in April 2021 and the significant reduction in social restrictions from May 2021, when controlling for confounding factors, mental health outcomes in the CEV cohort were significantly worse than the non-CEV group. Although unlikely to be attributable solely to stringent social distancing itself and likely to be a combination of lower baseline mental health scores pre-pandemic, the increased psychological burden of disease during the pandemic, fear of contracting the disease and inconsistent public health messaging, these results highlight the potential detrimental role that stringent social isolation might have on this population and the need for post-pandemic mental wellbeing support in this group [21].

Physical health declined across the cohort. The physical health in the CEV group was, as expected, lower than that in the non-CEV group at all time points, and the outcome physical health scores were significantly worse in the CEV group compared with non-CEV group. This is likely to be attributable to an interplay of factors, including worse pre-pandemic health in the CEV group and less contact with health-care services that are relied upon more in the CEV group; in addition, these data indicates that in addition to mental health, stringent social distancing measures are likely to contribute to a worsening in physical health [22]. Rheumatological disease is a risk factor itself for worse physical functioning, and maintaining physical health in RD patients is important to combat the effects of the condition [23, 24]. Therefore, these data suggest that rheumatology departments should target physical health in their patients, particularly those classed as CEV, post-pandemic to help resolve these impacts.

### Infection rates and sequalae

Despite the extra precautions in the CEV patient group and consistently more CEV patients reporting self-isolation throughout the pandemic, infection rates in the CEV and non-CEV patients were similar. Vaccination rates were exceptionally high in both groups, and there was no difference between the groups in symptoms or duration of infection. We did not see an increased risk for long COVID symptoms in our CEV group, with ∼12% of our cohort experiencing long COVID symptoms through the period. Differences in infection rates in the CEV group could be influenced by reduced exposure to COVID-19 because of social isolation, varying degrees of contact with health-care professionals, and, potentially, increased testing rates, increased frailty and immunosuppressive therapy that define the CEV group. Accepting these limitations, there were no differences in infection in these groups.

### Use of mobile technology for research and dissemination

Our patients received SMS-based electronic video-based advice on vaccination and shielding in March 2020 and December 2020 outside of this research; subsequent self-reported vaccination uptake in our cohort was very high (99%) and, as demonstrated in these data, more patients in the CEV group that received the shielding advice were observing more stringent social isolation than the non-CEV group [13, 14]. The questionnaire response rates and the acceptance of vaccination and isolation advice delivered via this method in this population adds strength to and demonstrates the utility of this methodology in delivering important health-care messages. In addition, this highlights the potential role for mobile SMS-based research recruitment and participation in patients with RD.

### Limitations

This study has several limitations. The response rate initially was 21.4%, and based on previously published departmental data, this study cohort was slightly older (mean age between 61 and 63 years) than our general rheumatology patient cohort (mean age 57.5 years) [25]. Despite inviting our entire patient cohort to participate, the response rate in non-White groups was disproportionately low, and further exploration is required to understand why this was the case. Furthermore, there was a significant participant dropout rate between questionnaires 1 and 4, with 56.4% responding to the final questionnaire; this altered the demographics of our final cohort, with a greater proportion being >60 years of age (57.3 *vs* 50.5%) and, as might be expected, fewer non-CEV participants (52.8 *vs* 53.5%) than in the initial cohort recruited in questionnaire 1. In addition, we acknowledge that those with poorer mental health might be more likely discontinue their study participation, which might artificially inflate the MCS scores of the cohort in subsequent questionnaires, and further work exploring this is required.

We lacked mobile phone numbers for patients across all our age groups. Anyone lacking access to a smartphone or internet technology, those with limited technology literacy and those unable to read English were not able to participate, introducing a response bias.

Although previous studies have demonstrated the suitability of the SF-12 questionnaire in assessing HRQoL in patients with rheumatic disease, investigations defining the minimally important difference in this population are lacking; therefore, although significant changes were found, these could not be confirmed as clinically meaningful [26].

Lastly, we had planned initially to measure mortality as a primary outcome; however, only 21.4% of patients completed the first questionnaire and fewer completed both questionnaires 1 and 2, as it is unlikely this loss to follow up is wholly due to COVID-19 mortaility rates we considered this insufficient to comment adequately on this.

### Strengths

Our initial response rate of 21.4% is consistent with other studies [27]. Rather than excluding older patients using this methodology, we recruited more patients in our older age groups than younger ones, and we present data from non-inflammatory RD patients. Our recruitment across an entire cohort reduces social media network bias from other research, and we included our patients in the questionnaire design and piloting [28].

### Conclusion

In this single-centre longitudinal study spanning the duration of national restrictions, we have described the extent of the impact on physical and mental health of COVID-19 in a large RD cohort. We found consistently poorer mental health scores in our most vulnerable (CEV) patients, and although there were significant improvements in mental health in the entire cohort between April 2020 and December 2021, mental health outcomes were significantly worse in the CEV population. Physical health declined across the entire cohort, and those in CEV, younger age and ethnic minority groups were particularly impacted. We found no differences in infection rates or long COVID when comparing our CEV and non-CEV patients, despite additional precautionary measures in the CEV group. Our data represent important effects of a pandemic on patients with RD and might be helpful to health-care providers and policymakers when considering responses to a future pandemic. The merits of communicating information to patients and research recruitment through SMS messaging have been demonstrated and can be used in delivering important health-care communications in future.

## Supplementary Material

rkad009_Supplementary_Data

## Data Availability

Data will be made available upon reasonable request to the corresponding author.
