# Supplementary material for: The impact of the COVID-19 pandemic and stringent social distancing measures on health-related quality of life and COVID-19 infection rates in patients with rheumatic disease: a longitudinal analysis through the pandemic
Source: Rheumatol Adv Pract. 2023 Jan 16;7(1):rkad009. doi: 10.1093/rap/rkad009 (PMC9897299; doi:10.1093/rap/rkad009)
Supplement: rkad009_Supplementary_Data [file rkad009_supplementary_data.pdf]

Welcome to this survey exploring the impact of COVID 19 on rheumatology patients

Dear Rheumatology Patient,

We thank you for participating in our survey sent on 09.06.2021. In order to gain a clearer understanding of the impact of COVID-19 on our patients we are conducting a follow up survey as part of our COVID-19 Rheumatology Impact Surveillance Project.

The questionnaire will take approximately 10 minutes to complete and must be completed in one go. The questions are about how you have been managing during the COVID19 pandemic and the impact it has had on your mental wellbeing. We stress participation is completely voluntary.

Similarly to the previous questionnaire, we ask for your mobile phone number to allow us to link your responses to your healthcare record as this data will assist in managing your care safely.

* 1. I confirm that I have read and understood the participant information for the study:

☐ Yes

* 2. I have had the opportunity to ask questions and all my questions have been answered to my satisfaction:

☐ Yes

* 3. I understand that my participation is voluntary and that I am free to withdraw at any time, without giving any reason, and without my medical or legal rights being affected.

☐ Yes

* 4. I agree for my data collected in this study to be used for future research.

☐ Yes

* 5. I agree for my data collected during this study to be shared with other members of the rheumatology team and research staff at the royal Wolverhampton trust.

☐ Yes

* 6. I agree to participate in the above study.

○ Yes

○ No

* 7. I agree for my medical records to be linked with my survey answers for the study and I agree to provide my mobile number to do this.

- [ ] Yes

* 8. Please provide your mobile phone number below to allow us to link your responses to your medical record

Mobile Phone Number ____________________

* 9. Since the last questionnaire (sent 09.06.2021) have you been exposed to someone with definite or suspected COVID19 (co-workers, family, other):

- ○ Definite only
- ○ Suspected only
- ○ Both definite and suspected
- ○ Neither
- ○ Not Sure

* 10. Since the last questionnaire have you worked in any of healthcare, nursing/care home, or a school?

- ○ Yes
- ○ No

* 11. Since the last questionnaire have you worked in any of: (tick all that apply)

- [ ] Hospital inpatient
- [ ] Hospital outpatient
- [ ] Clinic outside hospital
- [ ] Nursing home/elderly care home or group facility
- [ ] School clinic
- [ ] Home healthcare
- [ ] Other healthcare facility

* 12. Have you ever interacted in person with patients with definite or suspected COVID19 since the last survey?

- ○ Definite only
- ○ Suspected only
- ○ Definite and suspected
- ○ Neither
- ○ Not sure

* 13. Since the last survey on 09.06.2021 have you been admitted to hospital?

- ○ Yes
- ○ No

* 14. How long were you in hospital for?

- <24 hours
- 1-2 days
- 3-7 days
- 1-2 weeks
- more than 2 weeks

* 15. Have you been tested for COVID 19 since 09.06.2021 (last survey)?

- Yes - positive result
- Yes - was told I have COVID but had a negative swab result (false negative)
- Yes - I've had a COVID test and tested negative
- No I have not been tested

* 16. Since the last survey have you been infected with COVID 19?

- Currently infected or Infected within the last 4 weeks
- Infected with COVID 19 within the past year
- I was infected with COVID on or before the 09.06.21 and reported this in the last questionnaire
- Not infected and no previous infection with COVID 19

* 17. How long did your symptoms last?

- <24 hours
- 1-2 days
- 3-7 days
- 1-2 weeks
- 2-3 weeks
- 3-4 weeks
- 4-12 weeks
- More than 12 weeks

* 18. Did you have the classic symptoms of high fever and persistent cough for several days?

- Yes
- No

* 19. What was your temperature in degrees C?

- Less than 37.5
- 37.5 - 37.7
- 37.8 - 38.0
- 38.1 - 38.4
- 38.5 - 38.9
- 39.0 - 39.5
- More than 39.5

* 20. If you had a cough did you have a persistent cough i.e. coughing for more than an hour, or 3 or more coughing episodes in 24hours?

- Yes
- No

* 21. Did you experience unusual fatigue?

- Yes
- No

* 22. Did you have a headache?

- Yes
- No

* 23. Did you experience unusual shortness of breath?

- Yes - mild
- Yes - significant
- Yes - severe
- No

* 24. Did you have a sore throat?

- Yes
- No

* 25. Did you have a loss of smell or taste?

◯ Yes

◯ No

* 26. Did you have an unusually hoarse voice?

◯ Yes

◯ No

* 27. Did you experience any unusual chest pain or tightness?

◯ Yes

◯ No

* 28. Did you have any unusual stomach pain?

◯ Yes

◯ No

* 29. Did you have any diarrhoea?

◯ Yes

◯ No

* 30. Did you have unusual strong muscle pain?

◯ Yes

◯ No

* 31. Did you have any confusion/disorientation/drowsiness?

◯ Yes

◯ No

* 32. Did you have a reduced appitite (skip meals)?

◯ Yes

◯ No

33. Are there any other important symptoms you want to share?

* 34. Where were you while you were infected?

- ◯ At home - have had COVID symptoms but did not attend hospital
- ◯ At home - attended the emergency department but was sent home i.e. not admitted to hospital
- ◯ At hospital with suspected or definite COVID

* 35. If you were in hospital did you require intensive care?

- ◯ Yes
- ◯ No

* 36. Did you require oxygen at hospital?

- ◯ Yes
- ◯ No

* 37. Other than hydroxychloroquine and sulfasalazine (adivised to continue during infection) did you continue any other DMARD medication while infected?

- ◯ Yes, I continued my DMARDs
- ◯ No, I stopped my other medications
- ◯ Not taking other DMARD medications

* 38. Did you continue biologic medicines while infected?

- ◯ Yes, I continued my biologic medicines
- ◯ No, I stopped my biologic medicines
- ◯ Not taking any biologic medicines

* 39. Did you continue your steroid medicines while infected?

- ◯ Yes, I continued my steroids while infected
- ◯ No, I stopped my steriods while infected
- ◯ Not taking regular steriod medicines

* 40. Since the last questionnaire did you stop the medications prescribed by the rheumatology department for a reason other than the COVID 19 infection?

- ◯ No - I have continued on the same medication
- ◯ Yes - I have changed my medication under the advice of my rheumatologist/ GP
- ◯ Yes - I stopped my medication due to another infection
- ◯ Yes - I chose to stop or alter my medication (please specify below)

Other. Yes - I chose to stop or alter my medications myself because ....

* 41. Have you been vaccinated for COVID-19?

- ◯ Yes
- ◯ No

* 42. How many doses of the vaccine have you had?

- ◯ One
- ◯ Two
- ◯ Three (3rd Primary / booster)

* 43. What was the main reason for you not having the vaccination?

- ◯ Not in an eligible age group
- ◯ Concern regarding pregnancy/ fertility
- ◯ Concern regarding side effects
- ◯ Other (please specify)

* 44. How much have you been self isolating or following social shielding advice over the past week?

- ◯ I have not left the house
- ◯ I rarely leave the house - when I do I have little interaction with others (exercise)
- ◯ I have to leave the house often and am in contact with other people (still working outside the house or using public transport)

* 45. Have you extended stringent social shielding due to personal concerns?

- ◯ Yes
- ◯ No

Section 7 - Your Health and Well-Being:

* 46. Since the last survery have you visited your GP due to mental health concerns?

- ◯ Yes
- ◯ No

* 47. In general, would you say your health is:

Excellent	Very Good	Good	Fair	Poor
◯	◯	◯	◯	◯

* 48. The following two questions are about activities you might do during a typical day. Does your health now limit you in these activities? If so, how much?

	Yes - Limited A Lot	Yes - Limited A Little	No - Not Limited At All
Moderate activities such as moving a table, pushing a vacuum cleaner, bowling, or playing golf:	◯	◯	◯
Climbing several flights of stairs:	◯	◯	◯

* 49. During the past 4 weeks have you had any of the following problems with your work or other regular activities as a result of your physical health?

	Yes	No
Accomplished less than you would like?	◯	◯
Were limited in the kind of work or other activities?	◯	◯

* 50. During the past 4 weeks were you limited in the kind of work you do or other regular activities as a result of any emotional problems (feeling depressed or anxious)?

	Yes	No
Accomplished less than you would like:	◯	◯
Didn't do work or other activities as carefully as usual:	◯	◯

* 51. During the past 4 weeks how much did pain interfere with your normal activities:

Not at all	A little bit	Moderately	Quite a bit	Extremely
◯	◯	◯	◯	◯

* 52. The next 3 questions are about how you feel and how things have been during the past 4 weeks. For each question, please give one answer that comes closest to the way you have been feeling. How much of the time during the past 4weeks:

	All of the time	Most of the time	A good bit of the time	Some of the time	A little of the time	None of the time
Have you felt calm and peaceful?	◯	◯	◯	◯	◯	◯
Did you have a lot of energy?	◯	◯	◯	◯	◯	◯
Have you felt downhearted and blue?	◯	◯	◯	◯	◯	◯

* 53. During the past 4 weeks how much of your time has your physical health or emotional problems interfered with your social activities:

All of the time	Most of the time	A good bit of the time	Some of the time	A little of the time	None of the time
◯	◯	◯	◯	◯	◯

54. Please provide your mobile phone number below to allow us to link your responses to your medical record, if you did this earlier there is no need to re-enter it here

Mobile Phone Number [ ]

Many Thanks!
You have completed the survey. We greatly appreciate your time and support in this study.
